# Incomplete Early Childhood Immunization Series and Missing Fourth DTaP Immunizations; Missed Opportunities or Missed Visits?

**DOI:** 10.5402/2013/351540

**Published:** 2012-08-14

**Authors:** Steve G. Robison

**Affiliations:** Immunization Program, State of Oregon, Oregon Health Authority, 800 NE Oregon Street, Suite 370, Portland, OR 97232, USA

## Abstract

The successful completion of early childhood immunizations is a proxy for overall quality of early care. Immunization statuses are usually assessed by up-to-date (UTD) rates covering combined series of different immunizations. However, series UTD rates often only bear on which single immunization is missing, rather than the success of all immunizations. In the US, most series UTD rates are limited by missing fourth DTaP-containing immunizations (diphtheria/tetanus/pertussis) due at 15 to 18 months of age. Missing 4th DTaP immunizations are associated either with a lack of visits at 15 to 18 months of age, or to visits without immunizations. Typical immunization data however cannot distinguish between these two reasons. This study compared immunization records from the Oregon ALERT IIS with medical encounter records for two-year olds in the Oregon Health Plan. Among those with 3 valid DTaPs by 9 months of age, 31.6% failed to receive a timely 4th DTaP; of those without a 4th DTaP, 42.1% did not have any provider visits from 15 through 18 months of age, while 57.9% had at least one provider visit. Those with a 4th DTaP averaged 2.45 encounters, while those with encounters but without 4th DTaPs averaged 2.23 encounters.

## 1. Introduction

Timely immunization receipt is commonly assessed by up-to-date (UTD) rates for a combination of vaccine series, such as the 4 : 3 : 1 : 3 : 3 : 1 series recommended between birth and 18 months of age by the American Academy of Pediatrics (AAP) and the Advisory Committee for Immunization Practice (ACIP). The 4 : 3 : 1 : 3 : 3 : 1 series contains 4 DTaP (diphtheria/tetanus/pertussis), 3 IPV (polio), 1 MMR (measles/mumps/rubella), 3 HBV (hepatitis B), 3 Hib (*Haemophilus influenzae*), and 1 varicella vaccines. The need for four early DTaP immunizations is due to the continual reoccurrence of pertussis in the US, its risk to infants, and the difficulty in building adequate disease protection to pertussis [1]. Children who are not complete for their early childhood shots generally lack only one visit or shot, and the commonly missing component is the fourth DTaP due between 15 and 18 months of age [2, 3]. The lack of a fourth DTaP frequently holds down series UTD rates even when all other vaccines are well delivered. The UTD rate for completing four DTaPs as measured by the National Immunization Survey by 19 to 35 months of age lags behind most other early childhood immunizations [4]. Immunization UTD rates are used as measurable proxies for the overall receipt of appropriate early medical care and well-child visits [5]. The DTaP series by itself is also recommended as a proxy measure for receipt of all other early childhood immunizations [6]. As the only shot directly due by schedule at the 1.5-year-old well-baby visit, a lack of a timely fourth DTaP may imply a lack of appropriate well-child visits and services in this period.

The purpose of this study is to assess whether children who miss a timely fourth DTaP are also missing timely provider encounters during the 15- to 18-month period or are failing to have immunization eligible visits during this period. A challenge that this study addresses is that the usual data sources used to assess immunization UTD rates such as surveys or immunization registries typically do not contain information on provider visits where immunizations were not delivered. “Missed opportunities” are defined in immunization evaluation as a visit where some but not all recommended immunizations were delivered. However, visits where no recommended immunizations are delivered are invisible to the usual methods of calculating missed opportunities and UTD rates. In order to address this problem, the present study used a joined set of immunization records from Oregon's ALERT Immunization System (ALERT IIS), and medical encounter records for children enrolled in the Oregon Health Plan, so that children who missed a timely 4th DTaP could be assessed against their full provider visit record. Secondary questions for this study include whether having a usual source of care or medical home is related to higher rates of fourth DTaP completion and if the number of provider encounters during the 15- to 18-month period is also associated with more fourth DTaP receipt. 

## 2. Methods

Immunization records for two-year olds in 2007 were extracted from the Oregon ALERT IIS and joined to broader medical encounter records and enrollment information from the Oregon Health Plan (OHP). The OHP is administered by Oregon's Division of Medical Assistance Programs (DMAP) and consisted during the study period of traditional Medicaid populations and an expanded SCHIP population of children in families below 185% of the federal poverty level. OHP children are primarily placed into commercially managed healthcare plans in Oregon. Health plans and providers are required to submit detailed encounter records on OHP-enrolled children for all services received. All OHP children are eligible for free vaccines through the Vaccines for Children (VFC) program. The ALERT IIS receives immunization records from 95% of Oregon private healthcare providers seeing children and from 100% of public providers. 

From the merged dataset, a study population of children was selected for those eligible to receive a fourth DTaP according to the ACIP schedule at 15 to 18 months of age, and for whom provider encounter records during the 15- to 18-month period were expected to be submitted to the OHP. Possible confounders to testing the study question include the effects of parental vaccine hesitancy and gaps in either Medicaid or private insurance coverage [7]. To minimize the effect of such confounders, the study population was limited to children who had a record of timely receipt of their first three DTaP immunizations and a known public insurance source with free vaccine availability covering the period of 15 to 18 months of age. Specific selection criteria included having received a third DTaP by nine months of age according to ALERT; not having an early fourth DTaP prior to 15 months of age; active OHP enrollment covering the 15 to 18 months of age period when the fourth DTaP is due. 

Potential vaccination eligible encounters that occurred between 15 and 18 months of age were counted for members of the study population, based on a review of International Classification of Diseases (ICD-9) and Current Procedural Terminology (CPT) coding in OHP encounter records. A potential vaccination-eligible encounter was defined as an encounter occurring in a nonemergent or noninpatient setting with a medical provider eligible to deliver immunizations, and with either a CPT procedure code for routine care or evaluation, or a principle ICD-9 diagnostic code indicating that the purpose of the encounter was consistent with routine care and immunization evaluation. A medical home measure was created by counting the number of providers in ALERT who administered the first three DTaP doses to each child, so that those who received all of their prior DTaPs from a single source were defined as having a consistent medical home or usual source of care.

## 3. Results

**Figure 1:**
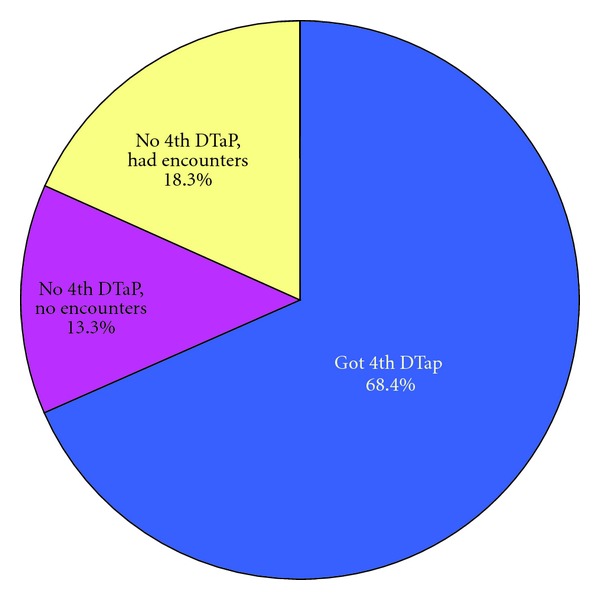
4th DTaP receipt between 15 through 18 months of age for children with 3 DTaPs by 9 months. Source: Oregon ALERT IIS, Oregon Immunization Program.

The study population consisted of 9,539 children who met the criteria of having three DTaPs by nine months of age, no early fourth DTaP, and OHP enrollment between 15 through 18 months of age. The study population represents 47% of the 2005 birth cohort with any length of enrollment in the Oregon Health Plan and 20% of the total Oregon 2005 birth cohort. Overall 8,113 children, or 86.7% of the study population, had at least one vaccination-eligible provider encounter between 15 and 18 months of age. A fourth DTaP was received between 15 and 18 months of age by 7,547 children, or 68.4% of the study population. These results are presented in Figure 1.

Of the study population, 13.3% did not have vaccination eligible encounters between 15 and 18 months of age, while 18.3% had vaccination-eligible encounters without receiving a fourth DTaP. Of those children who did not receive a fourth DTaP, 57.9% had at least one missed opportunity for the fourth DTaP between 15 and 18 months of age. 

**Figure 2:**
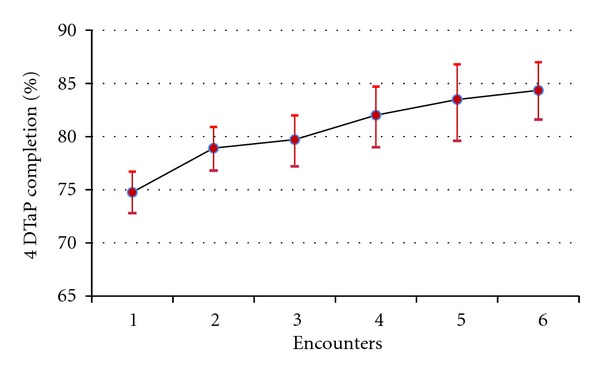
Third-to-fourth DTaP completion rates by number of provider encounters from 15 through 18 months of age. Source: Oregon ALERT IIS.

Among those who received a timely 4th DTaP, the average number of provider encounters between 15 and 18 months of age was 2.45 (95% c.i. of 2.42 to 2.49). Among those who did not receive a 4th DTaP but who also had at least one encounter, the average number of encounters was 2.22 (95% c.i. of 2.16 to 2.30). The chances of receiving a 4th DTaP significantly increased for those with two encounters (average rate of 78.9%, 95% c.i. of 72.8% to 76.7%) versus those with one encounter (average rate of 74.8%, 95% c.i. of 76.8% to 80.9%). Otherwise, while the trend was for increasing 4th DTaP receipt with increasing numbers of encounters, the differences were not statistically significant (Figure 2). 

The majority of the study population also presented evidence of having a usual source of care. For 91% of the study children, ALERT records contained sufficient information to identify a primary provider by clinic for each of their first three DTaP doses. The remaining 9% included at least one DTaP report from secondary sources such as billing or administrative data that could not be tied to a specific clinic. Of those with identified providers for all of their first 3 DTaPs, 76.1% had only a single provider, 8.1% had two providers, and 15.8% had three providers. However, as presented in Table 1, there was no significant difference in fourth DTaP rates between those with only a single provider versus those who used two or three providers. 

## 4. Discussion

The principal finding of this study was that the majority of children who missed a timely fourth DTaP had provider encounters between 15 months and 18 months of age that were potentially eligible for vaccine administration. This result was observed in a population where cost, access, and parental vaccine hesitancy were not substantial barriers due to the design of the study. Parents in this population were compliant with immunization recommendations for the first three DTaPs; and due to their enrollment in the OHP, access to vaccines without cost was assured. Prior work with this population has demonstrated that provider encounters without immunizations are also common across a larger age span from birth to the age of two [8]. In addition, one other study linking immunization registry and medical record data systems has shown similar problems of special population children visiting providers but not receiving needed immunizations [9]. The number of encounters children had during the period when the fourth DTaP was due was only mildly associated with their receipt of the 4th DTaP. While 75% of the study population with only one encounter received a fourth DTaP, the rate increased slightly to 84% for those with six or more encounters. This is consistent with prior findings that missed opportunities generally stay missed and are not readily caught up [10]. The small increases in UTD rate for more than one visit may indicate that providers were not routinely checking for missed shots beyond the first visit in the period, or that missed 4th DTaPs for reasons such as illness or parental preference will not be made up in subsequent visits. It is possible that a substantial number of 4th DTaPs were missed due to illness at the time of the provider encounter, even if the encounter was for a well-baby check. In a prior survey of parents, a child's illness was the most cited reason for delaying receipt of DTaP immunizations [11]. Similarly, provider reluctance to give immunizations during sick visits is a recognized barrier to timely immunizations [12]. As a measure of medical home, in this study there was no evidence to suggest that children with only a single source of immunizations were more likely to receive a fourth DTaP. This result varies from at least one previous finding that increases in the number of providers in early childhood were related to lower fourth DTaP rates [13]. As the 4th DTaP is the only immunization routinely due at 15 to 18 months, it is likely that most of the children not receiving a 4th DTaP in this study would be classified by usual methods of assessing immunizations based solely on the immunization record as lacking provider visits. This could potentially misdirect intervention efforts to focus solely on bringing children in for more encounters. In this study, the majority of the children without a 4th DTaP had one or more timely encounters where an immunization likely could have been delivered. 

There were several limitations to this study. The definition of immunization-eligible encounters based on a routine evaluation code in the medical record was broad and includes sick visits in various medical settings, with potential bias by site. However, mild illness with or without fever, such as for the most common reasons for sick visits including Otitis Media, is not valid contraindication to giving a fourth DTaP. Additionally, the study population was selected specifically to look at missing fourth DTaP immunizations, instead of any missing immunization, and so the results may not be generalizable to other vaccines. The study design was selected to reduce potential biases such as lack of access to care or previous reluctance to receive DTaP. Completion of other missing vaccines, such as the third polio dose, may be influenced by other factors, such as hesitancy to receive many injections at a time on each visit. Children with access barriers or whose parents opt for alternate vaccination schedules also will likely possess different patterns of immunizations at 15 to 18 months. The OHP population used in this study, while a large component of all Oregon infants, contains subgroups with higher medical and social risks which may influence provider and parent behavior. Findings may therefore not be generalizable to all Oregon children and point to the need for further analysis on the relation of provider type, setting, and reason for visit to the observed patterns of encounters without immunizations.

## 5. Conclusion

This study demonstrates the value of linking immunization data with medical encounter data. Improving immunization series rates in early childhood depends on taking advantage of existing opportunities to give missing DTaP immunizations, along with ensuring that children have appropriate and timely encounters for immunizations. 

## Figures and Tables

**Table 1:** Number of providers for first three DTaPs by fourth DTaP receipt.

Number of providers	*N*	4th DTaP	
		Rate	95% C.I.
1	6,456	69.4%	(68.3% to 70.5%)
2	685	68.6%	(65.0% to 72.1%)
3	1,340	69.9%	(67.1% to 72.1%)
